# Can We Improve Diosmetin Activity? The State-of-the-Art and Promising Research Directions

**DOI:** 10.3390/molecules28237910

**Published:** 2023-12-02

**Authors:** Monika Wujec, Marcin Feldo

**Affiliations:** 1 Department of Organic Chemistry, Medical University of Lublin, Chodzki 4a St., 20-093 Lublin, Poland; 2 Department of Vascular Surgery, Medical University of Lublin, Staszica 11 St., 20-081 Lublin, Poland

**Keywords:** diosmetin, new derivatives of diosmetin, biological activity

## Abstract

Diosmetin is a natural substance widely distributed in nature, with documented multidirectional biological effects. The wide spectrum of biological activity of diosmetin gives hope that derivatives of this flavonoid may also be used as drugs or dietary supplements used in many diseases. Modification of the structure may, on the one hand, lead to an increase in biological potency, new biological activity, or an increase in solubility and thus bioavailability. This is an important direction of research because the use of pure diosmetin is limited due to its low bioavailability. This work is an attempt to collect information on the possibility of modifying the structure of diosmetin and its impact on biological activity.

## 1. Introduction

**Scheme 1:**
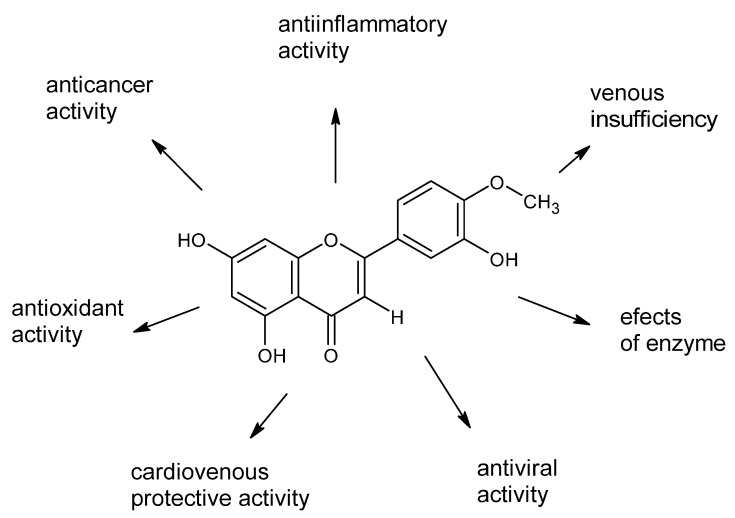
The overview of diosmetin activity.

Diosmetin is a natural flavonoid compound belonging to the flavone subclass of flavonoids. It is found in various plants and fruits including citrus fruits, such as oranges and lemons, and some herbs and various vegetables. *Rosmarinus officinalis* L., olives (*Olea europaea* L.), and lemon (*Citrus limon* L.) are sources of diosmetin with documented biological activity: anti-oxidant and cardioprotective activities [1,2,3,4,5,6]. Antioxidants are important for overall health and can play a significant role in preventing chronic diseases. Diosmetin is an aglycone of diosmin, a drug with a protective effect on blood vessels, used in the treatment of venous insufficiency. Over the last few years, interest in diosmetin has increased significantly. New activities were sought for this widely distributed flavonoid in nature. Diosmetin has been studied for its anticancer [7,8,9] and anti-inflammatory [10,11,12] properties. Moreover, its potential use in diseases associated with acetylcholinesterase (AchE) and butyrylcholinesterase (BuChE) inhibition has been investigated [13,14]. It has been found to have a positive effect on the reconstruction of cartilage after surgery [15]. Diosmetin was tested for use in the treatment of periodontitis [16]. Tasdemir et al. desribed antitrypanosomal and antileishmanial activities of flavonoids [17]. Baixon suggests that diosmetin protects against lung damage caused by the H1N1 virus, suggesting its potential use in the treatment of influenza-related diseases [18]. This year, a patent was published indicating the possibility of using diosmetin in infections with the SARS-CoV-2 virus [19]. The main directions of biological activity of diosmetin are shown in Scheme 1.

Unfortunately, the effect of diosmetin is often limited due to its physicochemical properties and, consequently, pharmacokinetic properties. The solubility of this flavonoid in an aqueous solution is low unless a very high pH is used, even in the presence of dissolution aids. In addition, bioavailability is usually low and permeation is thought to occur only after intestinal metabolism [20]. Scientists propose new forms of administration to improve solubility and, consequently, bioavailability. Solid SMEDDS (self-microemul-sifying drug delivery system) was prepared through electrospray by Gu et al. [21]. The use of this procedure allowed for the obtaining of a preparation with better solubility and bioavailability of diosmetin after oral administration. Russo et al. conducted a study to evaluate the pharmacokinetic profile of μSmin^®^Plus, a complex of micronized diosmin flavonoids standardized to diosmin and formulated with a buffering agent. The results were compared with the use of unformed micronized diosmin. Because µSmin^®^Plus was quickly and well absorbed into the systemic circulation, it may be a new preparation for use in interventional studies on humans [22].

Moriwaki and co-workers formulated diosmetin-7-glucoside-γ-cyclodextrin (DIOSG-CD) and compared the bioavailability of diosmetin after oral administration of DIOSG-CD with diosmin in Sprague Dawley (SD) rats. The study revealed that it presented inclusion, better solubility, higher bioavailability, and shorter absorption time than after diosmin intake in rats [23].

Other scientists have introduced diosmetin into liposomes [24]. Based on the research conducted, it was found that the new form possessed higher bioavailability and much-prolonged circulation time in rats compared with free diosmetine. A higher concentration of diosmetin in liposomes in the brain was also found, which may translate into a possible use in Alzheimer’s disease. To improve the hydrophobicity of diosmetin, the complex with lecithin was prepared by Brad et al. [20] The conducted research did not answer whether diosmin in complex with lecithin is characterized by better bioavailability.

**Figure 1:**
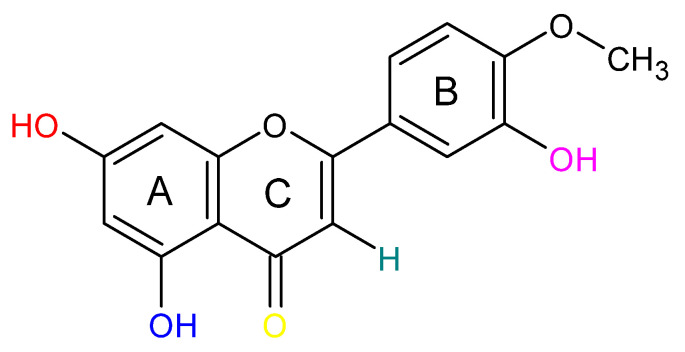
The places of modification of diosmetin structure.

In this work, we present a review of the literature on the possibility of modifying the structure of diosmetin to obtain biologically active compounds. We assume that the substrate for the synthesis was diosmetin and that a necessary element of the structure of the final compounds was the skeleton marked in black in Figure 1. The modification sites are marked with colors.

## 2. New Diosmetin Derivatives and Their Biological Activity

### 2.1. Anticancer Activity

**Figure 2:**
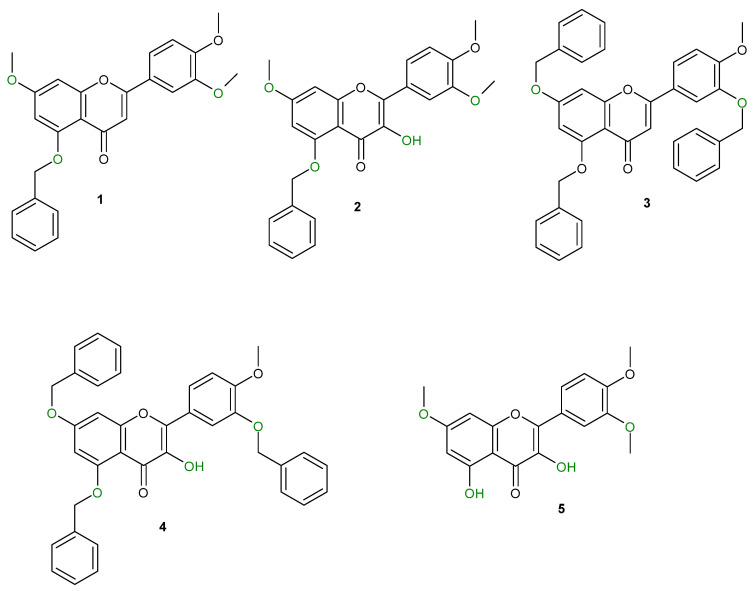
The most active benzyloxy derivatives of diosmetin.

Xie Jizhao et al. described synthetic procedures and biological investigations of some new flavonoid derivatives [25]. The benzyl-protected flavonol aglycone was synthesized through selective methylation, oxidation, benzylation, and other reaction steps. Then, the glycoside condensation reaction was carried out with the peracetylated bromo sugar and, finally, the flavonoid glycoside was synthesized by the reaction of debenzylation and acetyl group. A total of 32 flavonoids were synthesized, including 14 flavonoid aglycones and 18 flavonoid glycosides. Some of them were diosmetin derivatives (Figure 2).

All compounds were screened for anticancer activity using the breast cancer cell line SUM 149. The results showed that compounds **1** and **2** were 2-fold more active than **3** and **4**. The IC_50_ values were 5.23 μM, 5.64 μM and 9.34 μM, 9.65 μM, respectively. Compound **5** exhibited strong growth inhibitory effects on human triple-negative breast cancer cells, with an IC_50_ value of 1.38 μM.

In the next patent, the same authors described the anti-inflammatory activity of the previously obtained compounds [26]. Derivatives with benzyloxy substituent were active and had inhibitor activity on LPs-induced NO release from a macrophage RAW264.7.

Ferte et al. [27] obtained 28 flavonoid derivatives with a *N*-benzylpiperazine chain. New compounds were tested for their ability to modulate multidrug resistance (MDR) in vitro.

The authors classify the obtained compounds into the following three groups: derivatives of 7-(*N*-benzylpiperazinyl)flavones, 5-(*N*-benzylpiperazinyl)flavones, 3′-(*N*-benzylpiperazinyl)flavones and 7-(*N*-benzylpiperazinyl)flavanones, -chalcone, and dihydrochalcone. At a concentration of 5 µM, most of the tested compounds were able to increase the cytotoxicity of doxorubicin on the K/562/DOX cell line, which is a parental human erythroleukemia cell line obtained by stepwise selection with doxorubicin. This suggests that these compounds have the potential to overcome drug resistance in these cells.

The compounds were also able to increase the intracellular accumulation of JC-1. JC-1 is a fluorescent molecule used as a probe for P-glycoprotein-mediated multidrug resistance (MDR). The increase in JC-1 accumulation indicates that these compounds may act, at least partially, by inhibiting the activity of P-glycoprotein, which is known to be involved in drug resistance. The presented study found that lipophilicity played a role in modulating multidrug resistance (MDR) activity. However, lipophilicity alone was not the only factor determining the effectiveness of these compounds in overcoming drug resistance.

**Figure 3:**
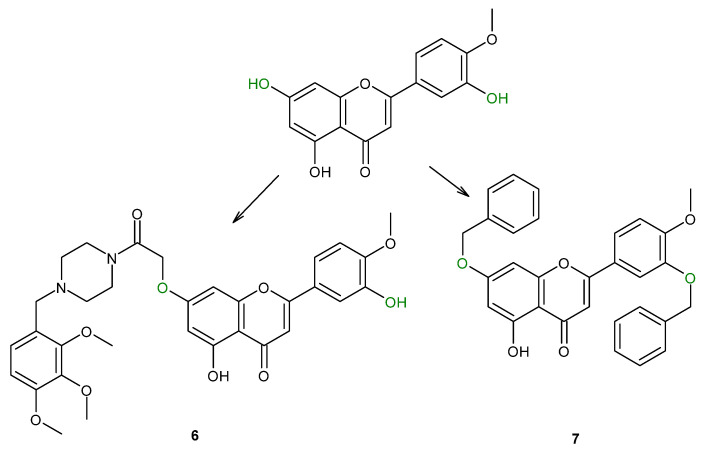
The most effective diosmetin derivatives.

The study examined various di- and trimethoxy substitutions on *N*-benzyl and observed that different substitutions had varying effects on the activity of these compounds. This suggests that the specific chemical structure of the compounds, including the nature and position of these substitutions, influences their ability to modulate drug resistance. Replacing the flavon ring with flavanone did not change the cytotoxic activity but reduced the toxicity. The most effective compounds identified in the study had a 2,3,4-trimethoxybenzylpiperazine chain or benzyl attached to either a flavone or a flavanone moiety. These compounds were more potent in overcoming drug resistance than verapamil, a known P-glycoprotein inhibitor. Two of them were diosmetin derivatives (Figure 3).

In summary, this study indicates that certain compounds have the potential to enhance the effectiveness of doxorubicin and overcome drug resistance in K562/DOX cells, possibly by inhibiting P-glycoprotein activity. Lipophilicity and the chemical structure of the compounds play important roles in their ability to modulate multidrug resistance.

**Figure 4:**
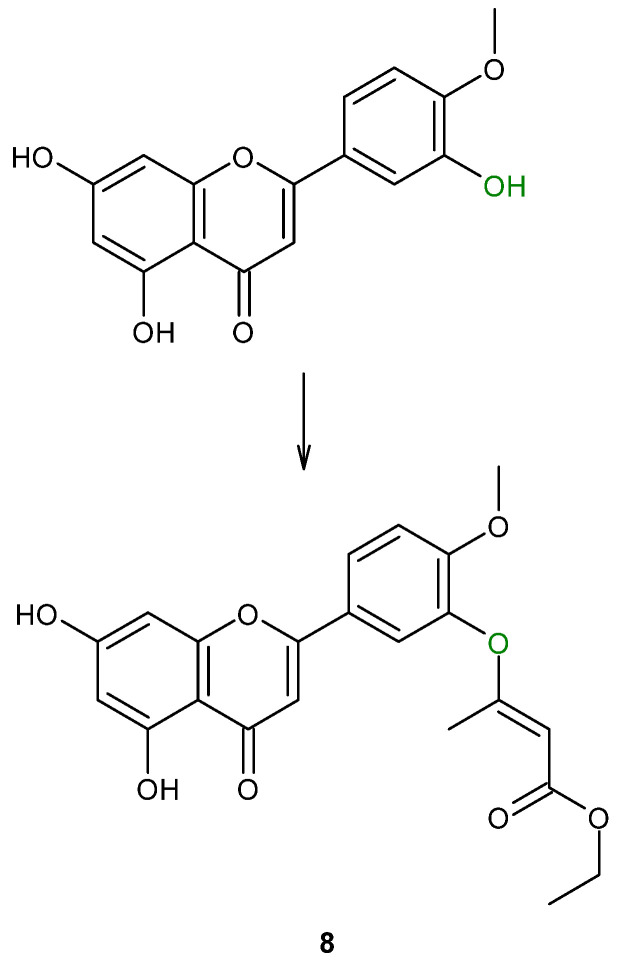
The structure of compound **8**.

Lu Jin et al. [28] proposed a method to enhance the suitability of flavonoids for drug development by modifying their chemical structure. Despite their potential therapeutic benefits, many flavonoids have not been developed into clinical drugs. One of the main challenges is their poor bioavailability, which can limit their effectiveness when administered as drugs. The researchers proposed a strategy to overcome the bioavailability issue by modifying flavonoids. They achieved this by constructing C(sp2)-O bonds and selectively adding alkenyl groups to hydroxyl groups on flavonoid molecules using ethyl-2,3-butadienoate allenes. This chemical modification is designed to enhance the drug-ability of flavonoids. The study involved the design, synthesis, and evaluation of 23 modified flavonoid derivatives. These derivatives are chemically altered versions of the original flavonoids. The anticancer potential of all compounds was evaluated using human leucemia cell lines K562 and HEL, prostate cancer line PC3, and non-small cell lung cancer A549. Several of the modified compounds showed better in vitro inhibitory activity against various cancer cell lines compared to their precursor flavonoids. Diosmetin derivative **8** (Figure 4) was less active than its precursor. Preliminary studies into the structure–activity relationship of these modified flavonoids suggested that the substitution at position 7 of the flavonoid molecule is crucial for increasing cytotoxicity against cancer cells.

**Figure 5:**
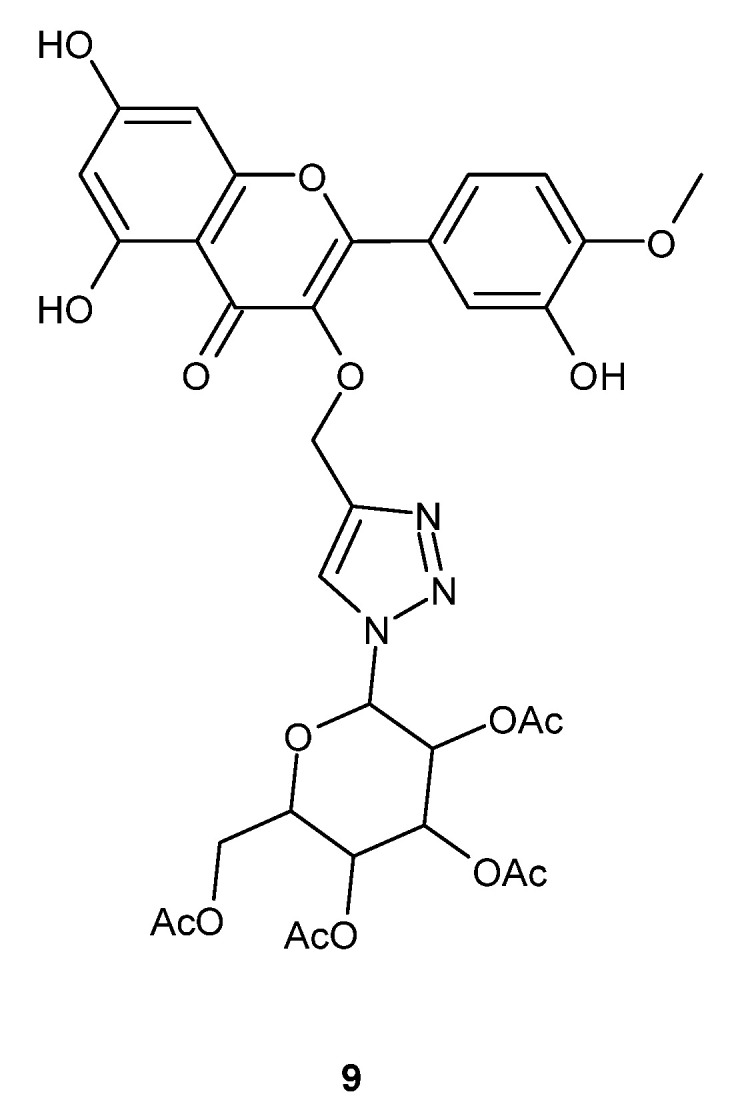
The conjugate diosmetin with 1,2,3-triazole and acylated galactose (**9**).

Lou, Dinghui Wang et al. [29] described the synthesis of 5 flavonoids derived from diosmetin and rhoifolin through a series of chemical reactions involving *O*-methylation, dehydrogenation, glycoside hydrolysis, *O*-benzylation, and dimethyldioxirane (DMDO) oxidation. These flavonoids are: 5,7,3′,4′-tetramethoxyflavonol, 5,7,3′-tribenzyloxy-4′-methoxyflavonol, 5,7,4′-trimethoxyflavonol, acacetin, and 5,7-dihydroxy-4′-benzyloxyflavone. The novel flavonoid galactoconjugates were synthesized using copper-mediated 1,3-dipolar cycloaddition reactions. These reactions involved acetylgalactose azides and alkynyl-substituted flavonoids. The synthesized compounds, including flavonoids and flavonoid galactoconjugates, were evaluated for their biological activities against five human cancer cell lines using the MTT method. The results of the biological activity evaluation showed that three compounds exhibited moderate cytotoxicity against five different human cancer cell lines, including HL-60, SMMC-7721, MCF-7, SW480, and A-549. The diosmetin derivative **9** (Figure 5) was characterized by lower activity than the derivative, and methoxy groups replaced hydroxyl groups.

**Figure 6:**
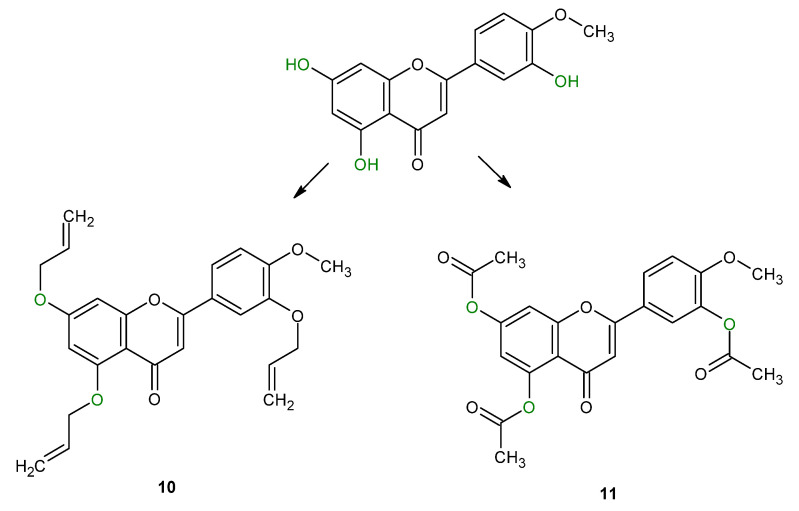
The most active molecules **10** and **11** derived from diosmetin.

T Kim-Dung Hoang published the synthesis and results of biological activity investigations of O-alkyl and O-acyl flavonoid derivatives [30]. The reagents used in the reactions were dimethylsulfate and allyl bromide. The authors state that the alkylation of the 5-OH group in the flavonoids depends not only on the alkylating reagent used but also on the structure of ring B in the flavonoids. The synthesized compounds were evaluated for their biological activities. Two specific tests were conducted: inhibition of bovine serum albumin denaturation and anti-inflammatory activity. In vitro tests assessed the ability of the compounds to inhibit the denaturation of bovine serum albumin. Denaturation of proteins can be a sign of inflammation and other pathological processes. Some compounds that showed significant inhibition of albumin denaturation were further evaluated for their anti-inflammatory activity. The study revealed that certain flavonoids effectively reduced carrageenan-induced paw edema, indicating their potential as anti-inflammatory agents. In the next step, in vitro antiproliferative activity was tested in the estrogen-dependent breast cancer cell line MCF-7. The IC_50_ values for all compounds were higher than camptothecin, which was used as a reference drug. The most active was the derivative of hesperetin, with an IC_50_ value of 0.020 µM/mL whereas for the diosmetin derivative **10** and **11** (Figure 6) the IC_50_ values were 0.035 µM/mL and 0.190 µmol/mL, respectively.

The study suggested that acyl derivatives of diosmetin and hesperetin could be valuable templates for the development of new anti-inflammatory agents and potential anticancer drugs.

**Figure 7:**
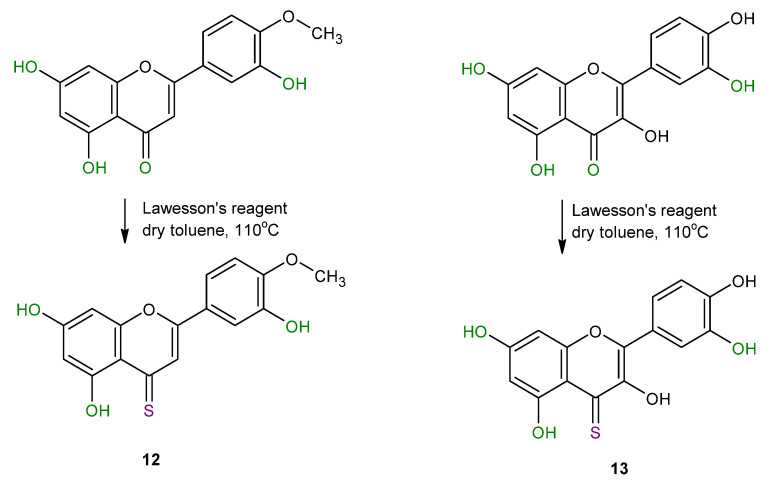
Synthesis of tioxoflavonoids derivatives **12** and **13**.

Wei Li et al. described the synthesis and antiproliferative activities of new flavonoid derivatives that have sulfur atoms instead of oxygen in C ring [31]. In the reaction with Lawesson’s reagent in anhydrous toluene, synthetic thioxoflavonoids were obtained. The synthesized thioxoflavonoids were tested for their ability to inhibit the proliferation of cancer cells in vitro. They exhibited moderate to good antiproliferative activities against three human cancer cell lines: Hela, HCC1954, and SK-OV-3. Generally, replacing a carbonyl group with a thio (sulfur) group enhanced the antiproliferative activity of the compounds. This suggests that the presence of sulfur in the molecules may contribute to their effectiveness against cancer cells. Among the thioxoflavonoids synthesized, three polymethoxy thioxoflavonoids and one polyhydroxy thioxoflavonoid were identified as promising candidates for further optimization and development as anticancer agents. Diosmetin analog **12** (Figure 7) was less active towards Hela and ovarian cancer cell lines. but was characterized by the same activity as a **13** to the epithelial breast cancer cell line.

The authors concluded that the antiproliferative mechanisms of thioxoflavonoids may be related to their antioxidant activity. This could involve their ability to scavenge free radicals or affect key redox enzymes such as thioredoxin, catalase, and GPx. These specific compounds demonstrated particularly favorable antiproliferative properties and are worthy of further study and development for potential medical use.

### 2.2. Effect on Enzymes

**Figure 8:**
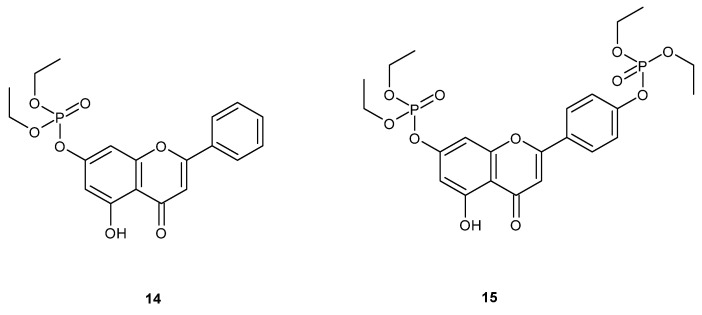
The most active phosphorylated compounds inhibiting the activity of carboxylesterases.

**Figure 9:**
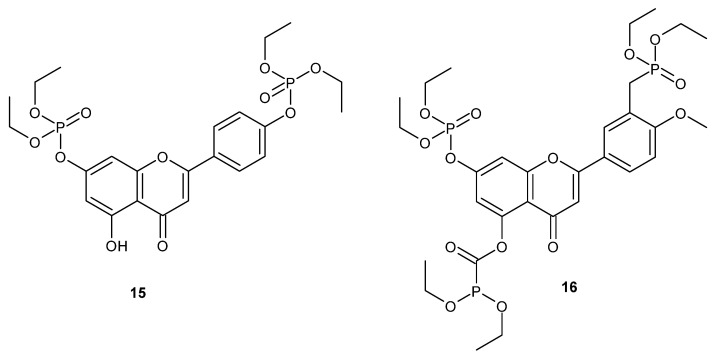
Diosmetin derivatives **15** and **16** with good selectivity towards CEase.

Wei et al. synthesized a series of flavonoids that were chemically modified with phosphorylation. The phosphorylated flavonoids were tested for the inhibition of carboxylesterases [32]. This is a family of enzymes responsible for the metabolism and detoxification of many xenobiotics containing an ester moiety. Some of these phosphorylated flavonoids were identified as potent inhibitors of pancreatic cholesterol esterase (CEase). CEase is an enzyme involved in cholesterol metabolism. CEase inhibitors can be used to limit the bioavailability of dietary cholesterol and, consequently, reduce its level in the blood [33]. The flavonoid derivatives showed excellent selectivity for inhibiting CEase over acetylcholinesterase (AChE). This selectivity is important because it indicates that these compounds target CEase specifically. The study also investigated the inhibitory activities of these compounds against porcine liver carboxylesterase (CE). Most active plant compounds are poorly absorbed by humans. The introduction of an ester group to the compound significantly improves its bioavailability. CE metabolizes compounds containing esters to the corresponding alcohols and carboxylic acids, which significantly affects their biodistribution [34]. The phosphorylated flavonoids exhibited significantly improved inhibition potency against CE compared to their parent compounds. Some of the phosphorylated flavonoids had a very high activity with IC_50_ values less than 5.0 nM. Two of them, compounds **3d** and **3e**, were identified as the most potent inhibitors of CE, with IC_50_ values of 1.79 nM and 1.58 nM, respectively. These compounds have one (**14**) or two (**15**) phosphorylated hydroxy groups (Figure 8). The diosmetin derived compound previously described as active (Figure 9) in the presented studies was ten times less active than the best-performing molecules. The study found that compounds with high inhibitory activities against CEase also efficiently inhibit CE. This suggests that the same structural features are important for inhibiting both enzymes. The presence of a free hydroxyl group at position 5 and a phosphate group at position 7 of the phosphorylated flavonoids was found to be favorable for the inhibition of CE. The most potent inhibitors were characterized as irreversible competitive inhibitors, indicating that they bind to the enzyme’s active site and cannot be easily displaced by the substrate.

The same authors in earlier work synthesized a series of new phosphorylated flavonoids [35]. These modified compounds were then tested for their ability to inhibit the activity of two enzymes, CEase and AChE. The results showed that most of the synthesized phosphorylated flavonoids exhibited nanomolar potency as inhibitors of CEase. Importantly, their inhibitory activity was much better than that of the parent flavonoids (flavonoids without phosphorylation). The phosphorylated flavonoids demonstrated good to high selectivity for CEase over AChE. In contrast, their inhibition of AChE was only in the micromolar potency range. This selectivity suggests that these compounds preferentially target CEase and are less active against AChE. The most potent inhibitor of CEase among the synthesized phosphorylated flavonoids was **15** (Figure 9). It had an IC_50_ value of 0.72 nM. This compound exhibited a remarkable 11,800-fold selectivity for CEase over AChE, highlighting its specificity for CEase. The derivative of diosmetin (**16**, Figure 9) was less active (IC_50_ = 20.6 nM towards CEase and 2940 nM towards AChE) but it was characterized by good selectivity. The SAR study revealed that the presence of a free hydroxyl group at position 5 and a phosphate group at position 7 of the phosphorylated flavonoids were favorable for the inhibition of CEase. This means that specific structural features in the modified flavonoids contributed to their potency against CEase. The inhibition mechanism of these phosphorylated flavonoids was also investigated. The results indicated that they act as irreversible competitive inhibitors of CEase. In competitive inhibition, a molecule competes with the substrate to bind to the enzyme’s active site, and in this case, the inhibition is irreversible, meaning it is long-lasting and not easily reversed.

Ning Cheng et al. described the synthesis of some derivatives of diosmetin, chrysin, luteolin, and apigenin [36]. Diosmetin derivatives were obtained in the reaction with bromoalkanes (ethyl bromide, butyl bromide, and hexyl bromide). The α-glucosidase inhibitory activity of these synthesized derivatives was tested, and their effectiveness was measured in terms of IC_50_ values. All of the synthesized derivatives showed significant α-glucosidase inhibitory activity, with IC_50_ values less than 24.396 µM/L. The most potent inhibitor among all tested compounds was O^3^′, O^7^-hexyl diosmetin with an IC_50_ value of 2.406 ± 0.101 mM/L whereas this parameter for references used was 563.601 ± 40.492 µM/L for acarbose and 226.912 ± 12.573 µM/L for 1-deoxynojirimycin.

**Figure 10:**
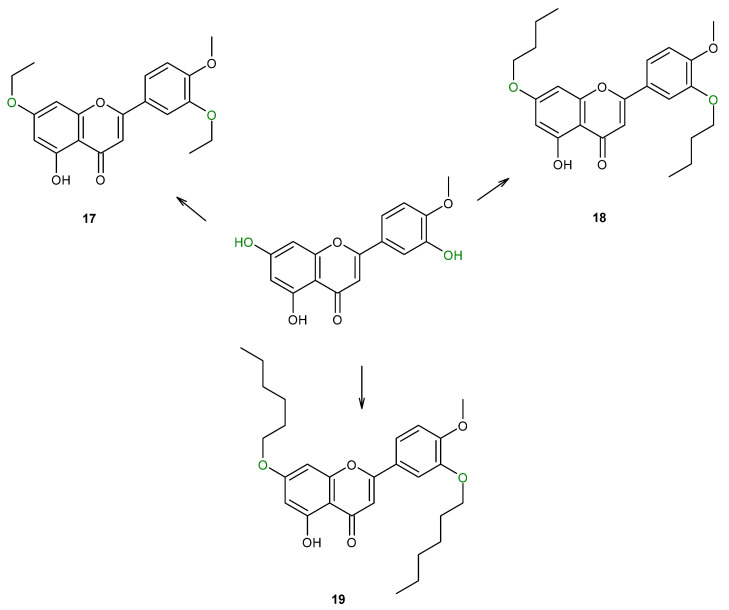
Derivatives of diosmetin (**17**–**19**) with good inhibitor activity.

Of the three diosmetin derivatives, two were characterized by high activity that was ten times higher than diosmetin. These were compounds with long-chain substituents (OC_4_H_9_ (**18**) and OC_6_H_13_ (**19**)) (Figure 10). The compound with an ethoxy substituent (**17**) had the same activity as diosmetin. The study indicated that the synthesized derivatives from natural flavonoids have the potential to be used as α-glucosidase inhibitors, which could be beneficial in managing blood sugar levels. Additionally, the length and position of alkyl chains on the flavonoids appear to impact their inhibitory activity.

The same flavonoid derivatives were synthesized by Nile et al. [37]. Diosmetin in the reaction with various bromoalkane (bromoethyl, bromobutyl and bromohexyl) in anhydrous acetone gave derived modification at the C-7 and C-30 positions (Figure 10). DPPH free radical-scavenging activity, ferric reducing/antioxidant power (FRAP)—reducing assay, and ORAC antioxidant assays were studied. Among the diosmetin derivatives, the compound with the longest alkyl chain (**19**) had the highest activity, while the derivative with two butyloxy groups had the lowest activity (**18**). None of the compounds showed higher antioxidant activity than diosmetin. Anti-inflammatory activities of newly synthesized flavonoid derivatives were determined using diene-conjugate, β-glucuronidase, LOX, and hyaluronidase in vitro inhibition assays. In these tests, the derivative with two hexyl chains (**19**) also had the highest activity; furthermore, activity was lower than that of diosmetins in this case.

**Figure 11:**
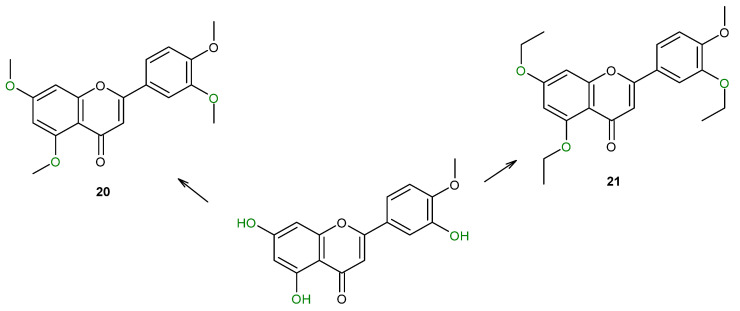
The structure of diosmetin derivatives **20** and **21**.

Natural substances such as flavones and flavonoids have different biological activities, including AChE and BACE-1 (Beta-secretase 1) inhibition, so they are attractive targets for Alzheimer’s disease. Thai-Son Tran et al. described synthesis, in silico, and in vitro evaluation of new flavone derivatives [38]. Two compounds derived from diosmetin **20** and **21** were the most active (Figure 11). The IC_50_ values were 73.82 µM and 75.91 µM for **20** and **21** derivatives for AChE and 5.78 µM and 5.80 µM for BACE-1, respectively. Comparing the activity of the obtained compounds to the reference drugs (Galanthamine and Umibecestat), it should be noted that they were characterized by significantly lower activity to both enzymes. However, it shows the direction of possible modifications and related biological activities.

Mannich base derivatives of flavonoids were synthesized in the reaction with dimethylamine, diethylamine, pyrrolidine, morpholine, piperidine, pipierazine, and formaldehyde in methanol with a good yield [39].

**Figure 12:**
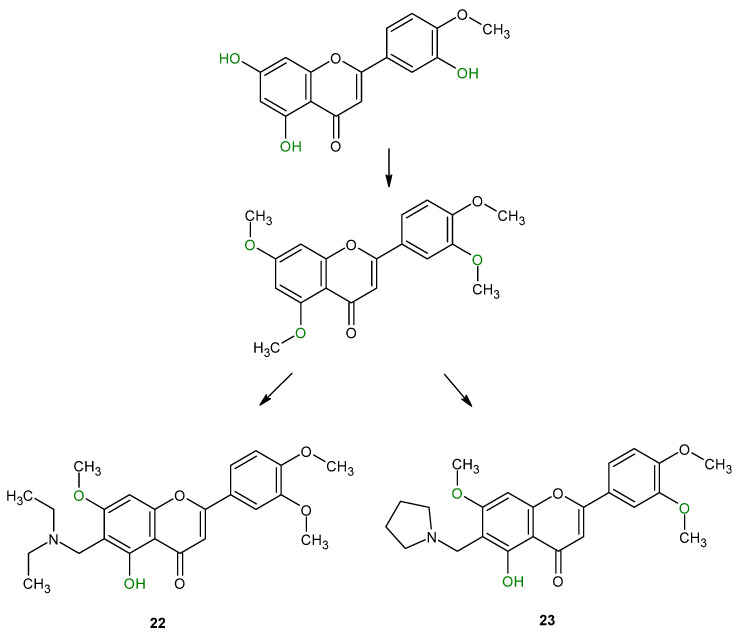
The scheme of the synthesis of diosmetin derivatives **22**–**23**.

The new compounds were characterized by spectral data. The reaction, when applied to a chalcone molecule, predominantly occurred at the C-3′ position of the B ring. This means that the reaction preferentially added or modified a chemical group at the C-3′ position on the B ring of the chalcone. When the same reaction was applied to a flavone molecule, it primarily took place at the C-6 position of the A ring. This indicates that the reaction had a preference for the C-6 position on the A ring of the flavone. All derivatives were screened for acetylcholinesterase inhibitory activities. The most potent were two compounds, **22** and **23,** with pyrrolidine and diethylamine moiety (Figure 12). The IC_50_ values of these were 0.54 µM/L and 1.39 µM/L, respectively, whereas the IC_50_ value for the drug neoeserine methyl sulfate was 1.38 µM/L. The authors concluded that alicyclic amine (piperidine) or N,N-dimethylamine improves the AChE inhibitory potential of the chalcones. In the case of flavones, the amines that promoted activity were N,N-diethylamine and pyrrolidine.

**Figure 13:**
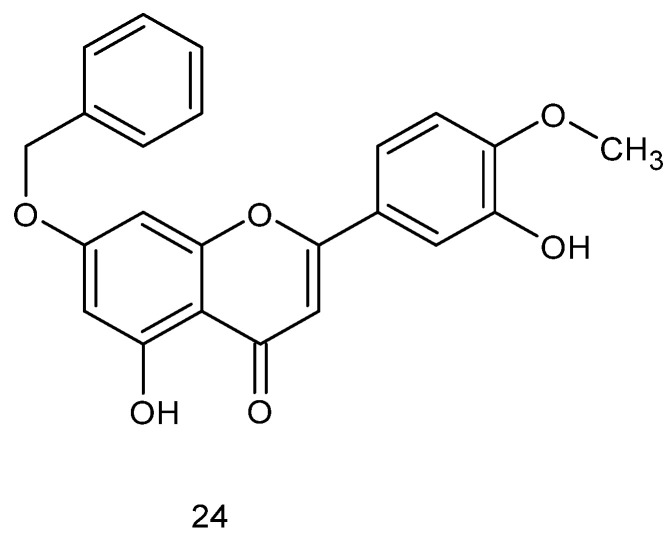
Benzil-substituted diosmetin (**24**).

Phosphodiesterase inhibitors are a class of agents approved for the treatment of hypertension, erectile dysfunction, chronic obstructive pulmonary disease, psoriasis, atopic dermatitis, and psoriatic arthritis [40]. Dhainanut et al. described the investigation of benzil-substituted diosmetin as a phosphodiesterase inhibitor [41]. Solipram was used as a reference drug. At the concentration 10^−7^ M the new compound **24** (Figure 13) inhibited enzyme activity by 60%.

### 2.3. Other Biological Acivities of Diosmetine Derivatives

A new class of anticoagulant agents was obtained by Correira-da-Silva et al. [42]. Polysulfated (oligo)flavonoids were synthesized using a method that involved molecular hybridization of two classes of anticoagulants, sulfated flavonoids, and sulfated polysaccharides. The new compounds were tested for their in vitro anticoagulant activities. Polysulfated flavonoids showed in vitro activity both in plasma and in whole human blood as well as in vivo, exceeding the anticoagulant activity of known flavonoids. Preliminary toxicity studies indicated the absence of acute side effects. Polysulfated diosmin was one of the most potent compounds. Polysulfated diosmetin was not tested due to insolubility. The SAR study revealed that 3-*O*-rutinosides were direct inhibitors of factor Xa (FXa), while 7-*O*-rutinosides showed inhibition of FXa by activating antithrombin III (ATIII). This information helps understand the mechanisms of their anticoagulant actions. The overall results of the study suggest that polysulfated flavonoids have the potential to be new and effective agents for anticoagulant therapy.

Xin Yang et al. described mechanical studies using both experimental and computational chemistry methods to determine the optimal pH, availability of oxygen, and counter-cations for efficient synthesis of flavonoid dimers and oligomers using the readily available monomeric flavonoids [43]. They employed luteolin coupling with diosmetin. Some of the newly obtained dimers and trimers showed good antibacterial activity. Dicranolomin and distichumtriluteolin inhibit the growth of *A. niger* with IC_50_ of 0.86 μM and 0.96 μM, respectively. The authors also performed tests for α-amylase and α-glucosidase inhibition. Some of the tested compounds were characterized by activity comparable to or higher than that of antidiabetic drugs.

Chinese authors have described methods for the synthesis of hydride compounds obtained from the combination of flavones with various quinoline derivatives [44]. Some of the obtained molecules showed good antibacterial activity against *Staphylococcus epidermidis*, *Cryptococcus neoformans*, and *Klebsiella pneumoniae*. However, there is no comparison of the activity of new compounds and antimicrobial drugs used with the quinoline system, e.g., fluoroquinolones.

**Figure 14:**
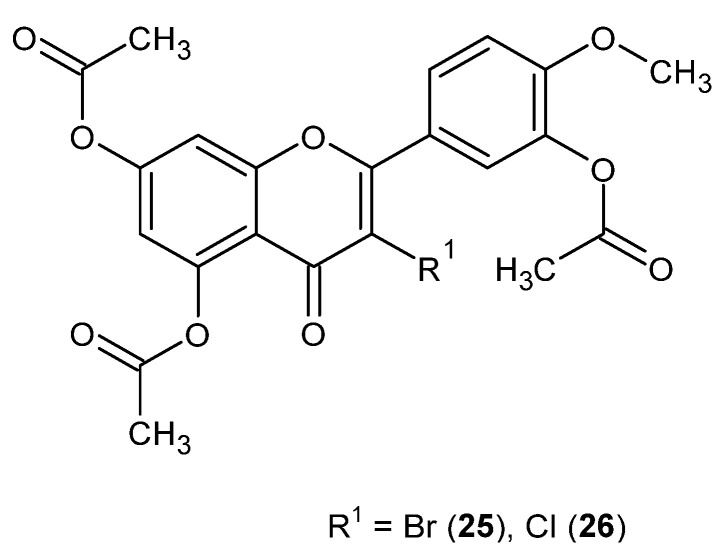
Structure of 7-substituted acylated diosmetin derivatives **25**, **26**.

Gay Lewin, in their patent, described the synthesis and antioxidant activity of new derivatives of diosmetin [45]. The author indicated that the introduction of chlorine and bromine atoms at position 7 of diosmetin increases the solubility in water and, consequently, the bioavailability. New compounds **25**–**26** (Figure 14) in a dose of 50–500 mg can be used for the prevention of diseases characterized by an excess of free oxygen radicals. such as cancer, ischemic diseases, dermatoses caused by UV radiation, neurodegenerative diseases, and symptomatic stages of venous insufficiency. However, comparative studies have shown that anti-free radical activity is lower than that of diosmetin.

**Figure 15:**
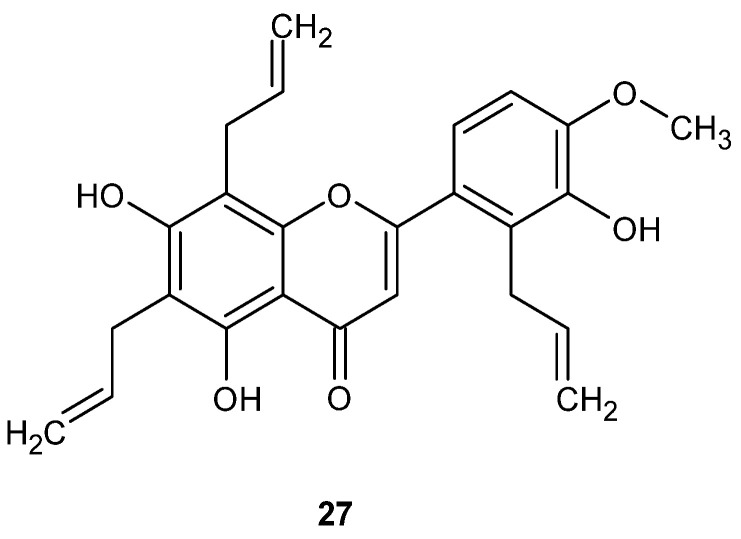
The structure of 6,8-diallyl-5,7-dihydroxy-2-(2-allyl-3-hydroxy-4-methoxyphenyl)-4H-1-benzopyran-4-one (**27**).

Wierzbicki et al. patented the structure of new derivatives of diosmetin and their anti-edematous activity [46]. The compounds were tested in a FITC-dextran extravasation procedure using tissue from hamster cheek pouches. The most active was 6,8-diallyl-5,7-dihydroxy-2-(2-allyl-3-hydroxy-4-methoxyphenyl)-4H-1-benzopyran-4-one (**27**) (Figure 15). The effect was observed to be dose-dependent.

**Figure 16:**
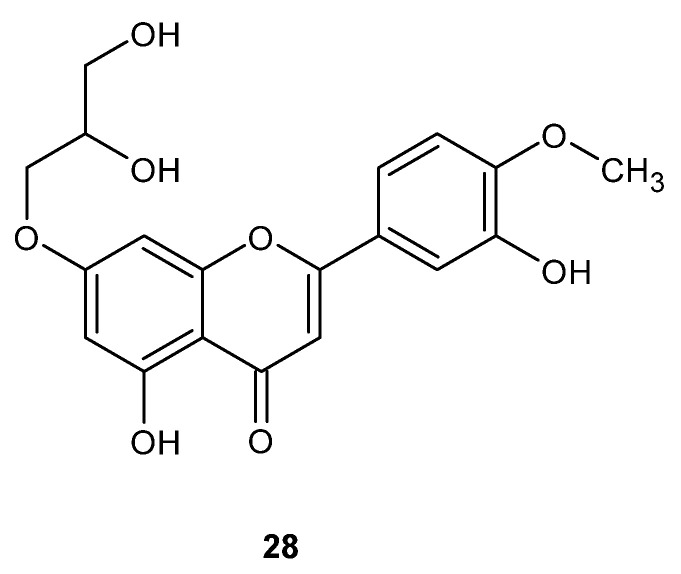
The structure of compound **28** (*R*,*S*) 5-hydroxy-2-(3-hydroxy-4-methoxyphenyl)-7-(2,3-dihydroxypropyloxy)-4H-1-benzopyran-4-one.

The obtained compounds were also tested for anti-inflammatory activity in a mouse model of auricle edema caused by external application of arachidonic acid. The evaluation criterion was the difference in the weight of the ear after using the acid and the test compound. Compound **28** (Figure 16) at a dose of 20 mg/kg b.w., used before smearing the ear with arachidonic acid, reduced inflammation by 20%. The authors did not use diosmetin as a reference substance, so it is impossible to say whether the modification increased activity.

## 3. Conclusions

As the review shows, diosmetin is still of interest to scientists as a leading structure in the basis for the search for new drugs. The syntheses focus primarily on compounds with potential anticancer activity. This is still one of the most current directions of research, which is related to the fact that cancer is the second cause of death in the world after cardiovascular diseases. Research into the preparation of compounds that may be used in Alzheimer’s disease also deserves special attention.

Based on the presented research results, it can be concluded that the modification in the B-ring of diosmetin promotes anti-cancer activity. Compounds with higher activity than the precursor were obtained. Acylation of hydroxyl groups in the A and B rings also allowed for the obtaining of new derivatives with high activity towards selected cancer cell lines. Replacing the oxygen atom with a sulfur atom also increased cytotoxic activity. Summarizing the enzymatic studies, it can be noted that phosphorylation of hydroxyl groups causes an increase in the inhibition of the activity of both CEase and AChE enzymes. The introduction of an amino moiety into the A ring of diosmetin also promotes activity towards AChE. It is also possible to combine two or more flavonoid molecules, which leads to compounds with antibacterial activity. Unfortunately, not all authors compare the obtained results of biological tests to the natural precursor, so it cannot be clearly stated that the modification of the structure increases biological activity. Undoubtedly, improving the solubility of diosmetin due to chemical modifications has a beneficial effect on its activity. The solubility can be increased by introducing substituents in the 7-position of diosmetin or by alkylation of the hydroxy group in the B ring of diosmetin. Further research into possible modifications of the structure of diosmetin is recommended.

Due to the discovery of new directions of diosmetin activity, it can be assumed that diosmin may also be used in the same diseases; however, further research is necessary in this case.

## Data Availability

Not applicable.
